# The Role of Physical Activity in Nonalcoholic and Metabolic Dysfunction Associated Fatty Liver Disease

**DOI:** 10.3390/biomedicines9121853

**Published:** 2021-12-07

**Authors:** Christian von Loeffelholz, Johannes Roth, Sina M. Coldewey, Andreas L. Birkenfeld

**Affiliations:** 1 Department of Anesthesiology and Intensive Care Medicine, Jena University Hospital, 07747 Jena, Germany; johannes.roth@med.uni-jena.de (J.R.); Sina.coldewey@med.uni-jena.de (S.M.C.); 2 Septomics Research Center, Jena University Hospital, 07747 Jena, Germany; 3 Center for Sepsis Control and Care, Jena University Hospital, 07747 Jena, Germany; 4 Department of Diabetology Endocrinology and Nephrology, Internal Medicine IV, University Hospital Tübingen, Eberhard Karls University Tübingen, 72074 Tübingen, Germany; Andreas.birkenfeld@med.uni-tuebingen.de; 5 Division of Translational Diabetology, Institute of Diabetes Research and Metabolic Diseases (IDM) of the Helmholtz Center Munich, Eberhard Karls University Tübingen, 72074 Tübingen, Germany; 6 Department of Diabetes, School of Life Course Science and Medicine, Kings College London, London WC2R 2LS, UK

**Keywords:** insulin resistance, type 2 diabetes, nonexercise activity thermogenesis, AMP activated protein kinase, ectopic lipids

## Abstract

Sedentary behavior constitutes a pandemic health threat contributing to the pathophysiology of obesity and type 2 diabetes (T2D). Sedentarism is further associated with liver disease and particularly with nonalcoholic/metabolic dysfunction associated fatty liver disease (NAFLD/MAFLD). Insulin resistance (IR) represents an early pathophysiologic key element of NAFLD/MAFLD, prediabetes and T2D. Current treatment guidelines recommend regular physical activity. There is evidence, that physical exercise has impact on a variety of molecular pathways, such as AMP-activated protein kinase and insulin signaling as well as glucose transporter 4 translocation, modulating insulin action, cellular substrate flow and in particular ectopic lipid and glycogen storage in a positive manner. Therefore, physical exercise can lead to substantial clinical benefit in persons with diabetes and/or NAFLD/MAFLD. However, experience from long term observational studies shows that the patients’ motivation to exercise regularly appears to be a major limitation. Strategies to integrate everyday physical activity (i.e., nonexercise activity thermogenesis) in lifestyle treatment schedules might be a promising approach. This review aggregates evidence on the impact of regular physical activity on selected molecular mechanisms as well as clinical outcomes of patients suffering from IR and NAFLD/MAFLD.

## 1. Introduction

The term nonalcoholic fatty liver disease (NAFLD) was defined in the 1980s to describe exceeding hepatocellular triacylglycerol accumulation in absence of significant alcohol intake, viral and autoimmune liver disease [1]. The course of NAFLD was long thought to follow the so-called “two hit hypothesis” [2]. Manifestation of bland steatosis (nonalcoholic fatty liver, NAFL) was defined as first hit, while signs of liver inflammation, hepatocyte injury and fibrosis, becoming evident in varying percentages of patients, were proposed as succeeding second hit. Presence of these pathologies can be evaluated histologically, using defined staging and grading systems, and is termed then nonalcoholic steatohepatitis (NASH) [1,3,4]. Steatosis can alternatively be evaluated by noninvasive approaches [4,5].

The prevalence of NAFLD is globally increasing and parallels the pandemic rise in obesity [6,7,8]. This is particularly apparent for subjects suffering from type 2 diabetes (T2D). A recent meta-analysis indicates that on a global perspective more than 55% of T2D patients suffer from NAFLD and a further 37% from NASH. Highest prevalences are reported for Europe, West Asia, and Pan-America [9]. Insulin resistance (IR) is related to hypertension and T2D and recognized as strongest predictive parameter of NAFLD progression, putting patients to an elevated risk of morbidity and mortality [4]. Sedentary behavior constitutes an associated risk factor and correlates with clinical outcomes [10,11]. It is accepted that lifestyle modifications including regular physical exercise can beneficially modify long term sequelae of prediabetes and T2D [12,13,14,15]. Therefore, regular physical activity is hypothesized to have effects on the prevention and amelioration of fatty liver disease. However, the majority of lifestyle interventions examined the impact of combined dietary and exercise strategies, while exclusive effects of physical training are less studied. This review will summarize available evidence concerning potential mechanisms and clinical benefits of exclusive physical training on ectopic lipid deposition. The scope will mainly cover the role of skeletal muscle, since it represents the organ system mostly affected by physical training. Furthermore, skeletal muscle has remarkable relevance in terms of whole-body insulin action and fuel homeostasis.

## 2. Insulin Resistance as Trigger Event for NAFLD Onset, Progression, and Clinical Course

NAFLD is recognized as a heterogenous disease, with disparate and complex causes of liver dysfunction [1]. Various maladaptations along with genetic influences are thought to be responsible for NAFLD onset and progression. The disease is therefore increasingly termed metabolic dysfunction associated fatty liver disease (MAFLD) [1]. Chronic subclinical inflammation and IR are considered as most significant molecular drivers of NAFLD/MAFLD progression [4,16,17]. Due to the potential risk of leading to liver fibrosis/cirrhosis this can determine patient prognosis [18,19,20,21,22,23]. NAFLD/MAFLD is related to renal and cardiovascular disease, whereby obesity and T2D are delineated as main pathologies linking NAFLD/MAFLD with long term sequelae [24,25,26,27,28,29]. Furthermore, metabolic fatty liver disease is a predictor of colorectal adenoma, related to the incidence of various malignancies, and in particular to the incidence of hepatocellular carcinoma (HCC) [21,22,30,31,32]. In the US, metabolic fatty liver disease is currently the second leading etiology of HCC-related liver transplantation and patients undergoing major surgery have more perioperative complications and longer hospital stay. After transplantation there is a significant risk of de novo T2D and NAFLD/MAFLD, and furthermore of premature death from cardiovascular complications and sepsis [33,34,35,36,37,38,39,40]. Together, MAFLD/NAFLD has to be considered a multisystem disease, as hepatic manifestation of the metabolic syndrome (MeSy)/T2D and can influence patient prognosis [39,41,42].

IR is the early pathophysiologic trigger event in the overnutrition-MeSy-T2D spectrum ([43]; reviewed in [44]). IR is closely related to ectopic lipid deposition, whereby liver fat is recognized as central predictor of whole-body insulin sensitivity, or reciprocally IR under human in vivo conditions [4,43,44,45,46]. The metabolic condition of skeletal muscle, the organ system most evidently impacted by physical exercise, was recently found to be influenced by liver lipid status in a dose dependent fashion [46]. Regular physical exercise can favorably modulate whole-body IR and improve glucose control and life expectancy of T2D subjects [47]. Therefore, guidelines on T2D management suggest regular physical activity as one causal treatment option [48,49]. This treatment strategy could also exert beneficial effects under conditions of NAFLD/MAFLD [50]. Current guidelines on the clinical management of metabolic fatty liver disease include such recommendations, although evidence is sparse and potential underlying molecular mechanisms are fragmentarily understood [51].

## 3. The Concept of Metabolic Flexibility: Molecular Mechanisms of Physical Activity on Glucose Metabolism and Insulin Signaling in Skeletal Muscle

The concept of metabolic flexibility is defined by the ability to rapidly adapt to conditional changes in energetic substrate demand, as for instance with transition from feeding to the fasted state, or acute onset of physical activity [52,53,54,55]. It was shown in the 1990s that postabsorpive IR humans expose markedly reduced skeletal muscle fatty acid oxidation, a state termed metabolic inflexibility [56]. Although evidence in this field has enormously grown in past decades it remains established that IR is a key component of metabolic inflexibility (reviewed in [54]). IR is clinically relevant mainly in white adipose tissue (WAT), skeletal muscle, and the liver. A broad body of evidence is available regarding the numerous molecular mechanisms responsible for the development of IR, to which the interested reader is referred [57,58]. Selected aspects in skeletal muscle, which can be modified by physical activity, will be in scope of this paragraph.

**Figure 1:**
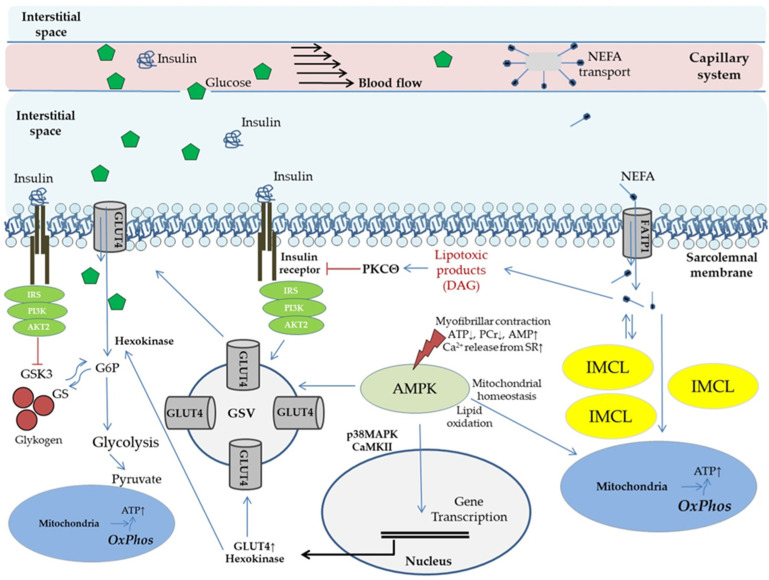
Potential molecular mechanisms of physical exercise and lipid species on glucose uptake and modulation of insulin action in skeletal muscle (conducted according to [57,63,64,66,67,68,69]). Physical exercise basically modulates supply of substrates and signaling molecules (via enhanced capillary perfusion, capillary recruitment/expansion of capillary volume); membrane transport of glucose (effects are majorly reported for GLUT4); mitochondrial adaptations (mitochondrial plasticity) and metabolic activation (glycolysis, lipid metabolism); and storage capacity and mobilization of energetic substrates (glycogen, IMCL). Effects of physical activity on insulin action and glucose uptake mediated by activation of AMP-activated protein kinase have been evaluated in various clinical settings (reviewed in [44]). AKT2, gene 2 encoding proteinkinase B; AMPK, AMP-activated protein kinase; CaMK, calcium/calmodulin kinase; DAG, diacylglycerol; FATP, fatty acid transport protein; G6P, glucose 6 phosphate; GLUT, glucose transporter; GS, glycogen synthase; GSK, glycogen synthase kinase; GSV, glucose transporter storage vesicle; IRS, insulin receptor substrate; IMCL, intramyocellular lipids; MAPK, mitogen-activated protein kinase; NEFA, non-esterified fatty acids; OxPhos, oxidative phosphorylation; PCr, phosphocreatine; PI3K, phospho-inositol 3 kinase; PKC, proteinkinase C; SR, sarcoplasmic reticulum.

Skeletal muscle accounts for 60–80% of insulin stimulation-mediated glucose metabolism [59]. Rising skeletal muscle metabolic activity by means of physical exercise therefore constitutes a promising therapeutic approach. Multiple mechanisms are discussed with regards to acute or chronic exercise training on insulin action and related substrate flux [60,61,62,63,64,65,66]. Figure 1 provides an overview.

**Figure 2:**
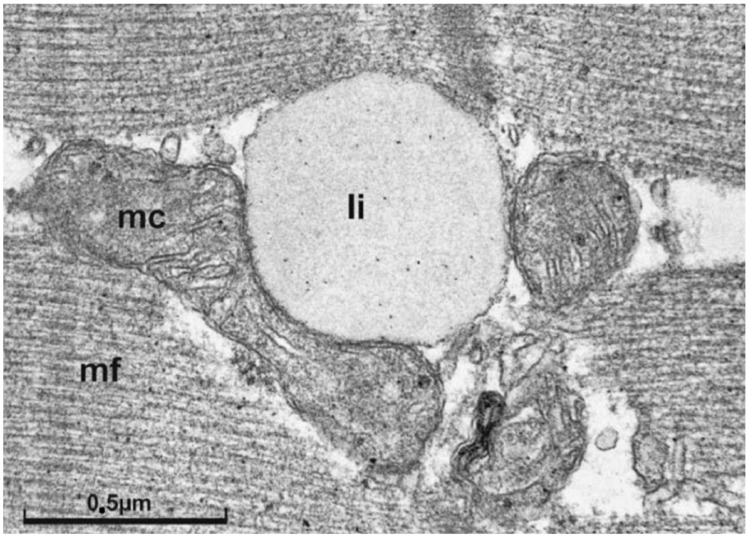
Electron micrograph of a longitudinal section of skeletal muscle tissue. In the center, at the z-line level, interfibrillar mitochondria with a lipid droplet immediately adjacent are shown (micrograph taken from [89] with kind permission of [90] and Springer-Nature). In support of the concept of metabolic flexibility it is believed that greater IMCL storage capacity in athletes represents an adaptive response to regular physical training, allowing a larger contribution of the local lipid pool as an energetic substrate source during exercise in order to preserve glycogen [89,91]. Li, lipid droplet; mc, central mitochondria; mf myofilament; marker indicates 0.5 µm.

Hallmarks of peripheral IR are impaired glucose transporter 4 (GLUT4) mediated glucose uptake by skeletal muscle, and compromised suppressibility of WAT lipolysis (reviewed in [44]). Visceral obesity is common in IR subjects and extensive evidence implicates that elevated circulating non-esterified fatty acids (NEFA) from inappropriate WAT hyperlipolysis are contributing to the etiology [17,70,71]. Under hyperlipolytic conditions increased proportions of NEFA are taken up by skeletal muscle. As indicated in Figure 1 this is followed by a rise in diacylglycerol (DAG), a product exerting potential lipotoxic effects on insulin signal transduction, resulting in reduced insulin stimulated GLUT4 translocation [17,64]. Supporting this, in vivo experimental settings in humans have shown that increased NEFA exposure of skeletal muscle reduces both, non-oxidative and oxidative glucose metabolism, as mirrored by 50% reduced glucose oxidation rates and glycogen storage capacity, respectively [58,72,73]. Furthermore, “NEFA overflow” under IR conditions is related to a rise in intramyocellular lipid (IMCL) deposition, which could play a role in buffering NEFA influx [58]. IMCL, particularly the depots in the subsarcolemmal region, correlate with the presence of IR under in vivo conditions in obese subjects, and are associated with cellular DAG and ceramide levels [74,75,76]. IMCL elevation is hypothesized to result not alone from increased lipid uptake, but also from impaired mitochondrial function. Experimental research supports this hypothesis, showing that skeletal muscle overexpression of the human catalase gene to mitochondria protects from age-related mitochondrial dysfunction and lipid-induced IR [77]. A role for mitochondrial dysfunction is further supported by findings in young lean and normoglycemic subjects with diabetic parents, exposing a 60% increase in IMCL along with a 38% reduced mitochondrial density, and 60% diminished insulin stimulated glucose uptake [78]. Knowledge on IMCL was just recently expanded by showing that contribution of IMCL to whole-body lipid oxidation could decrease in an obesity dependent manner [76]. Interestingly, lean old and young subjects had comparable IMCL, while old obese subjects had more than twofold greater IMCL and were more IR. The authors of this study suggest that skeletal muscle IR and lipid accumulation are likely due to lifestyle factors rather than inherent ageing of skeletal muscle [76]. Remarkably, normal weight endurance trained athletes also have higher IMCL levels with concomitantly increased muscle DAG, but are at the same time more insulin sensitive as compared to sedentary normal weight and obese subjects (“athlete’s paradox”) [74]. In that regard it is known that muscle contractions, comparable to insulin stimulation, can increase DAG levels in skeletal muscle cells and potentially play a role in adaptations induced by exercise [79,80,81]. Therefore, regular physical exercise could not alone normalize DAG related metabolism, but also impact specific proteins involved in subcellular IMCL formation and mobilization. Moreover, physical training appears to improve (or maybe even preserve) mitochondrial function, mediated at least in part by AMP-activated protein kinase (AMPK) [66,69,74,75]. The latter phenomenon is of specific interest in the discussion according to NAFLD/MAFLD, since it was shown by Michael Rodens’ group in humans in vivo that patients suffering from NASH have substantial mitochondrial dysfunction despite higher mitochondrial mass, resulting in impaired metabolic flexibility [82]. It is well known that IR correlates with mitochondrial function, even in skeletal muscle [83]. Otherwise, beneficial effects of regular exercise on mitochondrial plasticity are recognized [84,85]. For instance, rigorous physical exercise under specific conditions just over few weeks was lately shown to improve muscle mitochondrial volume density by as much as 50% and citrate synthase activity by 40% [86]. Consequently, mitochondrial dysfunction can be defined as central pathology related to IR and ectopic lipid accumulation, while physical activity can be interpreted as a potent treatment option to restore or at least preserve metabolic flexibility. Remarkably, not only endurance training, but also resistance exercise can exert favorable adaptions on ectopic lipid metabolism [87,88]. Figure 2 shows a representative IMCL droplet in skeletal muscle of a trained subject. The close spatial relationship of IMCL and mitochondria could be indicative of a “logistic adaptation”, to be able to quickly respond to increased substrate demand.

Another elementary energy storage substrate in terms of physical exercise is glycogen. There is an inverse relationship of IR-status, glycogen synthase activity and glycogen storage capacity in human skeletal muscle in vivo [72,92,93]. By contrast, exercise-induced depletion of depots is followed by an enhanced ability to synthesize glycogen [65]. Using a defined depletion-recovery protocol under combined exercise and dietary restriction conditions, followed by carbohydrate overfeeding over days resulted in glycogen storage capacity in humans as high as 15 g per kilogram body weight [94]. Glycogen depletion due to exercise and repletion by dietary intervention during recovery is a routinely used strategy of many athletes [95,96]. Moreover, highly trained endurance athletes can increase fatty acid oxidation in response to lipid overload. At the same time glycogen storage within muscle is preserved at the expense of decreasing glucose oxidation. This maneuver, which is associated with higher mitochondrial capacity of the exercised muscle, represents a unique example of metabolic flexibility [54]. As regards NAFLD/MAFLD, improved glycogen storage and mobilization capacity would be desirable, since this would theoretically help to relieve glucose load from the liver and thereby leave less substrate for de novo lipogenesis (reviewed in [44]). Remarkably, a single bout of exercise can substantially rise insulin sensitivity in IR subjects, while the subsequent increase in insulin stimulated skeletal muscle glucose uptake and glycogen synthesis can be observed for up to 48 h. Interestingly, glycogen levels are increased independently from muscle glycogen content under such conditions (reviewed in [96]). Further research is required to explore which preconditions and exercise schedules will result in best clinical results, specifically in ageing human NAFLD/MAFLD patients. However, it appears that exercising in the fasted state can substantially stimulate glycogen synthesis and IMCL breakdown, at least in young healthy volunteers [97]. Moreover, GLUT4 content of skeletal muscle is related to muscle mass, suggesting potential over-additive effects of a combined endurance and resistance exercise schedule [98].

Finally, one aspect in terms of exercise which can potentially result in unfavorable adaptations needs to be discussed. There is a known relationship between exercise intensity and improved glucose uptake [66]. However, very intense exercise (particularly eccentric exercise, i.e., downhill running), resulting in disruption of muscle cell integrity followed by delayed onset of muscle soreness due to eliciting local inflammatory response can decrease glucose disposal in skeletal muscle for up to 48 h (reviewed in [99]). Although earlier data suggest a compensatory pancreatic β-cell response resulting in raised insulin levels after eccentric exercise in young healthy subjects, it is still unclear whether this remains true for older IR patients [100]. Furthermore, it has been shown very recently that excessive training (i.e., high intensity interval training, HIIT) results in impaired mitochondrial function and glucose intolerance [101]. This clearly indicates that exercise schedules for improving insulin action, glucose uptake and ectopic lipid storage in older IR subjects require professional assessment, appropriate planning, monitoring and management.

Together, regular physical exercise can beneficially impact gross adaptational processes involved in fuel storage and mobilization associated with IR. The concept of metabolic flexibility provides some explanation governing fuel selection between NEFA and glucose, with the related substrate shift serving more efficient energy source utilization during exercise. Beyond this metabolic flexibility enables the switch from catabolic to anabolic processes in which energy substrates can be effectively stored after muscle activity [54]. These adaptations are realized by a multitude of modulations on the transcriptomic, proteomic, and epigenomic level. Obviously, AMPK appears to have a key regulatory function in this situation ([102,103]; reviewed in [54]). From a pathophysiological perspective the model of metabolic flexibility is specifically attractive under conditions of NAFLD/MAFLD, since it can be concluded from existing literature that the regularly exercised skeletal muscle provides substantial surplus storage capacity for energetic substrates (i.e., NEFA and glucose). Moreover, restoration of skeletal muscle fuel depots relies on provision from food sources, further contributing to relieve the liver from an overflow of potential nutritoxic substrates [104,105].

## 4. Data on Lifestyle Interventions under Conditions of Insulin Resistance and NAFLD/MAFLD

There is a plethora of data suggesting beneficial effects of lifestyle interventions on clinical endpoints in T2D [47,106]. Furthermore, in subjects suffering from prediabetes, i.e., impaired glucose tolerance (IGT), lifestyle intervention studies have proven to decrease the incidence of T2D (reviewed in [48]). Prominent examples comprise the DaQuing Study, the Finish Diabetes Prevention Study and the Diabetes Prevention Program [12,15,107,108]. Over a period of few years the risk of incident T2D was reduced by more than 50% in these trials. The latter is supported by a report on the Lifestyle intervention and Impaired glucose tolerance Maastricht (SLIM) trial, which has been shown to still impact clinical endpoints four years after stopping the intervention [109,110]. In contrast the almost 10-year lasting prospective randomized LookAHEAD study in T2D patients was unable to find substantial effects on cardiovascular events, although the intervention group experienced significant weight loss [111]. Of note, secondary analyses of this hallmark study were able to show that the magnitude of weight loss may be predicting in terms of outcome measures [112]. Mean weight loss in most intervention studies was modest, typically ranging around 5 kg or less. Thus, a variable percentage, yet not all the longer-term effects, can be apparently explained by weight loss. Together, there is decent evidence that physical activity as part of a lifestyle intervention schedule cannot alone impact metabolic control, but also beneficially influence outcome parameters in subjects suffering from IR, which has been recognized in recommendations on the clinical management of T2D [48,113].

What can we learn from these data when focusing on NAFLD/MAFLD? As outlined, IGT, T2D, and NAFLD/MAFLD have IR as a core pathophysiologic trigger event in common. Physical exercise is capable of improving and maybe reversing IR, and it is reasonable to assume that it represents a potent treatment modality in motivated patient groups. Short term decreases in physical activity coming along with a rise in sedentary behavior are sufficient to reduce multiorgan insulin-sensitivity and in parallel increase liver fat, supporting the given hypothesis [114]. Regarding that, improved insulin action in peripheral tissues is thought to represent the main mechanism contributing to liver fat decline following exercise (reviewed in [115,116]). However, only few data are available on this matter. Most studies have significant shortcomings as for instance (extremely) short duration, uncontrolled and/or non-randomized or retrospective design, and/or application of a combination of dietary intervention and exercise methods. However, all of them were more or less consistently able to show beneficial effects on primarily liver fat and most also on features of IR/glucose control [117,118,119,120,121,122,123,124,125,126,127]. Prospective randomized controlled studies exclusively examining the effects of regular physical training on NAFLD/MAFLD features are rare. Table 1 summarizes representative interventions using exercise-only approaches.

From the data presented in Table 1 it becomes evident that exercise is capable of consistently reducing liver fat in a restricted yet significant manner. Correspondingly a recent metaanalysis including studies with appropriate design showed significant effects of physical training on liver fat [116]. This effect was independent from changes in body weight, but the results suggest that the outcome will be more substantial under weight loss conditions [116,138]. Importantly both aerobic endurance and resistance exercise are capable of exerting beneficial adaptations on insulin signaling and therefore probably also on liver fat [48,139]. It should be otherwise kept in mind that after cessation of exercise any beneficial effects are lost within relatively short time periods, pointing to the importance of long-term compliance when aiming at promoting regular exercise as a serious treatment option for NAFLD/MAFLD patients [140].

In conclusion exercise without dietary intervention can reduce liver fat and exert positive mechanistic effects on insulin signaling. Such effects can become more prominent when combining exercise and dietary intervention in a lifestyle treatment-schedule to support weight loss. Of note, if regular exercise may positively modify features of NASH, e.g., inflammation, hepatocyte ballooning and fibrosis progression, remains momentarily unanswered due to lack of appropriate studies.

## 5. The Concept of Non-Exercise Activity Thermogenesis

**Figure 3:**
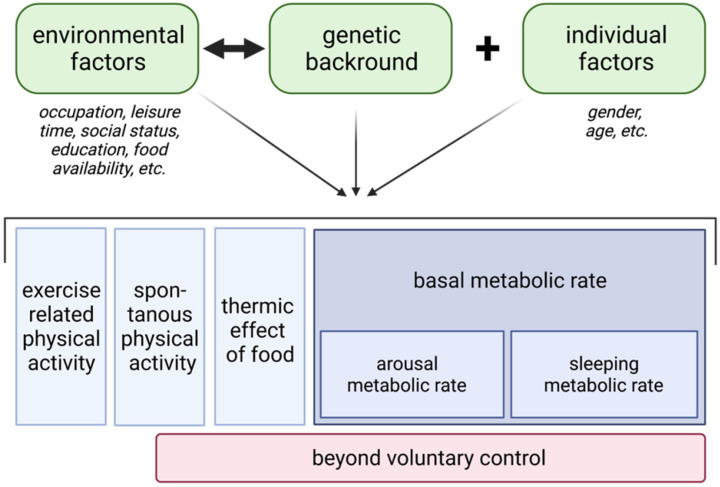
Model of human energy expenditure components (adapted from [141]).

Adherence represents a central precondition when aiming at treating NAFLD/MAFLD by means of physical exercise. NHANES data show that more than 36% of the studied US population is categorized as sedentary and a further almost 48% are physically active at low levels (reviewed in [141]). Only around 16% of subjects in NHANES met the recommended guidelines for physical activity or were considered to be highly active. Thus, it is reasonable to conclude, that from the perspective of a population level the percentage of subjects engaging in regular intense physical exercise is low. It can be hypothesized that without a supervised training schedule most patients under “real world conditions” will show poor compliance in the long term. The study of Pugh and colleagues indicates impressively that in absence of a supervised program beneficial effects of physical activity on NAFLD/MAFLD are quickly reversed [140]. The control groups in many of the trials in Table 1 further support this hypothesis, since control subjects were typically advised to be physical active, yet without supervision compliance was limited. Otherwise, it is recognized that everyday activity as for instance ambulation (walking) can exert significant effects on glucose metabolism in T2D. Seasonal impairment of glycemic control during wintertime due to lower physical activity levels and more sedentary behavior is well documented [142]. Available evidence does not support the hypothesis that a structured rigorous physical training has more pronounced effects on liver fat when compared to more moderate training sessions (see Table 1). Moreover, low to moderate physical activity is known to improve indices of IR, but under such conditions exercise duration needs to be considered an important factor when intending to impact insulin signaling in a relevant manner (reviewed in [143]). Given the limited participant motivation of most NAFLD/MAFLD or T2D patients it could be promising to encourage subjects to engage in more everyday activity. The energy expenditure related to such physical activity is known as non-exercise activity thermogenesis (NEAT). As can be taken from Figure 3, three main components of daily energy balance determine total energy expenditure (TEE), namely basal or resting metabolic rate, diet-induced thermogenesis (thermic effect of food), and physical activity-related energy expenditure (reviewed in [141,144]).

In western civilizations physical activity-related energy expenditure (PEE) accounts for maximally 30% of TEE in most individuals. PEE can be further categorized into exercise-related activity thermogenesis (EAT) and NEAT. These vary widely within and among subjects. EAT is defined as planned, structured, and repetitive physical activity aiming at improving health status, physical fitness, and quality of life. In those who habitually participate in purposeful physical training, EAT is believed to maximally account for 15–30% of daily energy expenditure (reviewed in [141]). In contrast, NEAT represents the predominant component of daily activity, which is also true for the majority of subjects undergoing regular physical training. NEAT is the “unnoticed” energy expenditure including energy expended for maintaining and changing posture (laying, standing), and other activities of daily living, which are not categorized as exercise training (i.e., walking, stair climbing, spontaneous muscle contraction). The significance of NEAT becomes apparent when considering the following points: The variability in basal/resting metabolic rate between individuals of similar age, BMI and of equal gender ranges around 7–9%, while the contribution of diet-induced thermogenesis is maximally 15% (reviewed in [141]). Thus, basal/resting metabolic rate and diet-induced thermogenesis are relatively fixed in amount and account for roughly three quarters of daily TEE variance. In contrast NEAT represents the most variable component (reviewed in [141]).

Note that parts of spontaneous physical activity are beyond voluntary control (i.e., “fidgeting”). Human overfeeding experiments shed interesting light on the importance of NEAT regarding its relevance for daily energy balance. Levine and coworkers were the first to systematically investigate the effect of overfeeding on the individual ability to adapt NEAT in free-living subjects (reviewed in [141]). By using sophisticated methods and measuring NEAT over a representative time span, they overfed volunteers by 1000 kcal day^−1^ in excess of their weight maintenance requirements. The energy surplus was paralleled by a mean rise in TEE of 554 kcal day^−1^. 336 kcal day^−1^ of the TEE increase was attributable to enhanced physical activity thermogenesis. Volitional exercise remained at a constantly low level and therefore about 60% of the increase in TEE due to overfeeding was attributable to NEAT. Fascinatingly NEAT adaptation varied remarkably between subjects, ranging from −98 to +692 kcal day^−1^ (reviewed in [141]). Due to the fact, that these findings were not consistently reproducible in later studies, it seems possible that variable inter-individual adaptations in thermogenesis by changes in NEAT are an explanation why some humans are susceptible to weight gain while others are not [141].

From the recent section it becomes clear that NEAT is principally capable of significantly impacting energy balance. A systematic review showed that subjects under a prescribed diet for weight loss may reduce daily activity in a compensatory manner, which could potentially contribute to body mass regain after cessation of the diet [145]. Otherwise, NEAT-related physical activity can be relatively easy integrated in daily patient routines (i.e., climbing stairs instead of using a lift, walking instead of using a car) and when exceeding a “critical” exercise volume and intensity (i.e., moderate to vigorous) it could potentially influence body weight and related metabolic features in a positive manner [136,146]. For instance, it was recently shown that increasing NEAT can contribute to improved postprandial lipemia and fat oxidation rates [147]. Whether this holds true for NAFLD/MAFLD conditions remains to be investigated. Since a sedentary lifestyle is otherwise not only associated with obesity, but also with various unfavorable clinical endpoints, increasing NEAT can be recognized as a promising way of lifestyle modification and should be regarded by future trials when considering physical activity as a treatment option. Such studies should also address the question whether increases in everyday activity is capable of positively impacting liver steatosis and IR/insulin sensitivity, and which volume (i.e., minutes day^−1^) and intensity (i.e., “NEAT activity with sweating” vs. “no sweating”) of exercise is necessary to observe beneficial effects.

## 6. Conclusions

Sedentarism is a worldwide pandemic and related to unfavorable clinical outcomes including premature mortality. Regular physical exercise can evidently impact features of IR, whole-body energy homeostasis and ectopic energy substrate depots in a health promoting manner. It is recognized that regular physical exercise can reduce the incidence of T2D and probably liver steatosis as one main pathophysiological feature of NAFLD/MAFLD. This beneficial impact is independent from body weight reduction, but effects are more pronounced under conditions of weight loss. Positive modulation of insulin signaling appears to represent the main responsible mechanism. One major limitation in terms of physical exercise as a treatment option remains patient motivation. Encouraging patients to engage in more everyday physical activity could be a promising strategy to overcome this problem. This hypothesis needs to be evaluated by future research.

## Figures and Tables

**Table 1:** Randomized controlled studies examining exclusive physical exercise effects in NAFLD/MAFLD.

Author	Design	Intervention and Methods	Outcomes	Drop Out
[128]	Randomized, placebo controlled n = 23 sedentary NAFLD/MAFLD patients	1 month supervised aerobic cycling exercise vs. stretching (placebo) IR (HOMA-IR), dietary record monitoring, visceral adipose volume, liver fat (^1^H-MR spectroscopy)	Significant reduction of liver fat and visceral adipose volume (intervention group) under conditions of unaltered dietary habits No effects on IR No effects on body weight	Drop out/excluded from analysis: n = 4 (17%)
[129]	Randomized, controlled n = 21 NAFLD/MAFLD patients	Partially supervised resistance exercise (2 months) vs. control Glucose control/IR (fsOGTT-AUC, HOMA-IR), liver lipids and abdominal fat (^1^H-MR spectroscopy), body weight	Significant reduction of liver fat (intervention group) Improved glycemic control and IR (intervention group) No effect on body weight and body fat	Drop out/excluded from analysis: n = 2 (9%)
[130]	Randomized, controlled n = 45 obese adolescent males	Supervised aerobic vs. resistance exercise vs. control (3 months) Insulin sensitivity (HE and HH clamp), liver fat (^1^H-MR spectroscopy, in subgroups), abdominal fat and body fat (whole-body magnetic resonance imaging)	Body weight stabilization (both intervention groups) compared to controls (weight gain) Significant reduction of liver fat and visceral adipose volume (intervention groups) Improved insulin sensitivity (resistance exercise group)	Drop out/excluded from analysis: n = 3 (7%)
[131]	Randomized, controlled, n = 33 NAFLD/MAFLD Patients	Partially supervised aerobic exercise vs. control (4 months) NAFLD/MAFLD-related Lipoprotein kinetics (tracer methods), body composition (DEXA), liver fat (^1^H-MR spectroscopy)	Significant reduction of liver fat (intervention group) No effect on body weight and body fat No effect on lipoprotein kinetics	Drop out/excluded from analysis: n = 15 (45%)
[132]	Randomized, controlled, n = 82 NAFLD/MAFLD Patients	3 months of partially Supervised resistance Exercise vs. placebo (stretching) Body composition (DEXA), dietary record monitoring, liver steatosis (HRI)	Significant reduction of liver fat, body fat and trunc fat mass (intervention group) under conditions of unaltered dietary habits	Drop out/excluded from analysis: n = 18 (22%)
[133]	Randomized, controlled n = 29 NAFLD/MAFLD patients	Partially supervised high intensity interval cycling (3 months) vs. control Glucose control/IR (fsOGTT-AUC, HOMA-IR), body composition (air displacement plethysmography), liver fat (^1^H-MR spectroscopy)	Significant reduction of liver fat (intervention group) Improved 2-h glucose, no effect on IR Body fat and body weight reduction (intervention group)	Drop out/excluded from analysis: n = 6 (21%)
[134]	Randomized, placebo controlled n = 48 sedentary NAFLD/MAFLD patients	2 months supervised aerobic cycling exercise (subgroups with varying volume and intensity) vs. stretching/self massage/ fitness ball (placebo) Dietary monitoring, visceral adipose volume (magnetic resonance imaging), liver fat (^1^H-MR spectroscopy)	Significant reduction of liver fat and visceral adipose volume (intervention group) under conditions of unaltered dietary habits	Drop out: n = 0 (0%)
[135]	Randomized, controlled n = 69 NAFLD/MAFLD patients	Supervised aerobic exercise (4 months) vs. counselling (control) Peripheral insulin sensitivity, dietary monitoring, hepatic glucose production (HE clamp in a subgroup), abdominal fat (magnetic resonance imaging), liver fat (^1^H-MR spectroscopy)	Significant reduction of liver fat (supervised exercise) (*p* = 0.05) under conditions of unaltered dietary habits Improved glycemic control and peripheral insulin sensitivity (supervised exercise) Body weight and abdominal fat mass reduction (supervised exercise)	Drop out/excluded from analysis: n = 19 (28%)
[136]	Randomized, controlled n = 220 NAFLD/MAFLD patients	6 months of vigorous- moderate exercise (jogging and brisk walking) vs. 12 months of moderate exercise (brisk walking) vs. control (no exercise) Liver fat (^1^H-MR spectroscopy after 6 and 12 month), body weight, waist circumference, body fat	Significant reduction of liver fat after 6 and 12 months (in both exercise groups) Reduced body fat (vigorous- moderate exercise group after 6 and 12 months) Reduced waist circumference (both exercise groups after 12 months) Reduced body weight (both exercise groups after 12 months)	Drop out/excluded from analysis: n = 9 after 6 months (4%), n = 14 after 12 months (6%)
[137]	Randomized, controlled, n = 26 sedentary NASH patients	Supervised combined aerobic and resistance exercise (3 months) vs. standard care (control) Body composition (air displacement plethysmography), glycemic control/IR (fsOGTT-AUC, HbA1c, HOMA-IR), circulating markers of liver fibrosis, liver fat (^1^H-MR spectroscopy)	Significant reduction of liver fat and visceral adipose tissue (intervention group) No effects on glycemic control or IR No effects on body composition No effects on circulatory markers of fibrosis	Drop out/excluded from analysis: n = 2 (8%)

DEXA, dual energy X-ray absorptiometry; fsOGTT-AUC, frequently sampled oral glucose tolerance test-area under the curve; HbA1c, glycated hemoglobin A1c; HE, hyperinsulinemic euglycemic; HH, hyperinsulinemic hyperglycemic; HOMA-IR, homeostasis model of insulin resistance; HRI, hepato-renal ultrasound index; IR, insulin resistance; MR, magnetic resonance.

## Data Availability

Not applicable.

## References

[1] Eslam M., Sanyal A.J., George J. (2020). MAFLD: A Consensus-Driven Proposed Nomenclature for Metabolic Associated Fatty Liver Disease. Gastroenterology.

[2] Day C.P., James O.F. (1998). Steatohepatitis: A tale of two “hits”?. Gastroenterology.

[3] Kleiner D.E., Brunt E.M., van Natta M., Behling C., Contos M.J., Cummings O.W., Ferrell L.D., Liu Y.-C., Torbenson M.S., Unalp-Arida A. (2005). Design and validation of a histological scoring system for nonalcoholic fatty liver disease. Hepatology.

[4] Stefan N., Häring H.-U., Cusi K. (2019). Non-alcoholic fatty liver disease: Causes, diagnosis, cardiometabolic consequences, and treatment strategies. Lancet Diabetes Endocrinol..

[5] Hannah W.N., Harrison S.A. (2016). Noninvasive imaging methods to determine severity of nonalcoholic fatty liver disease and nonalcoholic steatohepatitis. Hepatology.

[6] Kopelman P.G. (2000). Obesity as a medical problem. Nature.

[7] Bellentani S. (2017). The epidemiology of non-alcoholic fatty liver disease. Liver Int..

[8] Younossi Z., Anstee Q.M., Marietti M., Hardy T., Henry L., Eslam M., George J., Bugianesi E. (2018). Global burden of NAFLD and NASH: Trends, predictions, risk factors and prevention. Nat. Rev. Gastroenterol. Hepatol..

[9] Younossi Z.M., Golabi P., de Avila L., Paik J.M., Srishord M., Fukui N., Qiu Y., Burns L., Afendy A., Nader F. (2019). The global epidemiology of NAFLD and NASH in patients with type 2 diabetes: A systematic review and meta-analysis. J. Hepatol..

[10] Wen C.P., Wai J.P.M., Tsai M.K., Yang Y.C., Cheng T.Y.D., Lee M.-C., Chan H.T., Tsao C.K., Tsai S.P., Wu X. (2011). Minimum amount of physical activity for reduced mortality and extended life expectancy: A prospective cohort study. Lancet.

[11] Stamatakis E., Gale J., Bauman A., Ekelund U., Hamer M., Ding D. (2019). Sitting Time, Physical Activity, and Risk of Mortality in Adults. J. Am. Coll. Cardiol..

[12] Pan X.R., Li G.W., Hu Y.H., Wang J.X., Yang W.Y., An Z.X., Hu Z.X., Lin J., Xiao J.Z., Cao H.B. (1997). Effects of diet and exercise in preventing NIDDM in people with impaired glucose tolerance. The Da Qing IGT and Diabetes Study. Diabetes Care.

[13] Eriksson K.F., Lindgärde F. (1998). No excess 12-year mortality in men with impaired glucose tolerance who participated in the Malmö Preventive Trial with diet and exercise. Diabetologia.

[14] Tuomilehto J., Lindström J., Eriksson J.G., Valle T.T., Hämäläinen H., Ilanne-Parikka P., Keinänen-Kiukaanniemi S., Laakso M., Louheranta A., Rastas M. (2001). Prevention of type 2 diabetes mellitus by changes in lifestyle among subjects with impaired glucose tolerance. N. Engl. J. Med..

[15] Molitch M.E., Fujimoto W., Hamman R.F., Knowler W.C. (2003). The diabetes prevention program and its global implications. J. Am. Soc. Nephrol..

[16] Farrell G.C., van Rooyen D., Gan L., Chitturi S. (2012). NASH is an Inflammatory Disorder: Pathogenic, Prognostic and Therapeutic Implications. Gut Liver.

[17] Tilg H., Moschen A.R., Roden M. (2017). NAFLD and diabetes mellitus. Nat. Rev. Gastroenterol. Hepatol..

[18] Söderberg C., Stål P., Askling J., Glaumann H., Lindberg G., Marmur J., Hultcrantz R. (2010). Decreased survival of subjects with elevated liver function tests during a 28-year follow-up. Hepatology.

[19] Angulo P., Kleiner D.E., Dam-Larsen S., Adams L.A., Bjornsson E.S., Charatcharoenwitthaya P., Mills P.R., Keach J.C., Lafferty H.D., Stahler A. (2015). Liver Fibrosis, but No Other Histologic Features, Is Associated with Long-term Outcomes of Patients with Nonalcoholic Fatty Liver Disease. Gastroenterology.

[20] Ekstedt M., Hagström H., Nasr P., Fredrikson M., Stål P., Kechagias S., Hultcrantz R. (2015). Fibrosis stage is the strongest predictor for disease-specific mortality in NAFLD after up to 33 years of follow-up. Hepatology.

[21] Bertot L.C., Adams L.A. (2016). The Natural Course of Non-Alcoholic Fatty Liver Disease. Int. J. Mol. Sci..

[22] Kim G.-A., Lee H.C., Choe J., Kim M.-J., Lee M.J., Chang H.-S., Bae I.Y., Kim H.-K., An J., Shim J.H. (2018). Association between non-alcoholic fatty liver disease and cancer incidence rate. J. Hepatol..

[23] Hagström H., Nasr P., Ekstedt M., Hammar U., Stål P., Hultcrantz R., Kechagias S. (2017). Fibrosis stage but not NASH predicts mortality and time to development of severe liver disease in biopsy-proven NAFLD. J. Hepatol..

[24] Lu H., Liu H., Hu F., Zou L., Luo S., Sun L. (2013). Independent Association between Nonalcoholic Fatty Liver Disease and Cardiovascular Disease: A Systematic Review and Meta-Analysis. Int. J. Endocrinol..

[25] Than N.N., Newsome P.N. (2015). A concise review of non-alcoholic fatty liver disease. Atherosclerosis.

[26] Lee Y.-H., Kim K.J., Yoo M.E., Kim G., Yoon H.-J., Jo K., Youn J.-C., Yun M., Park J.Y., Shim C.Y. (2018). Association of non-alcoholic steatohepatitis with subclinical myocardial dysfunction in non-cirrhotic patients. J. Hepatol..

[27] Targher G., Byrne C.D. (2017). Non-alcoholic fatty liver disease: An emerging driving force in chronic kidney disease. Nat. Rev. Nephrol..

[28] Yeung M.-W., Wong G.L.-H., Choi K.C., Luk A.O.-Y., Kwok R., Shu S.S.-T., Chan A.W.-H., Lau E.S.H., Ma R.C.W., Chan H.L.-Y. (2018). Advanced liver fibrosis but not steatosis is independently associated with albuminuria in Chinese patients with type 2 diabetes. J. Hepatol..

[29] Buckley A.J., Thomas E.L., Lessan N., Trovato F.M., Trovato G.M., Taylor-Robinson S.D. (2018). Non-alcoholic fatty liver disease: Relationship with cardiovascular risk markers and clinical endpoints. Diabetes Res. Clin. Pract..

[30] Ma C., Zhang Q., Greten T.F. (2018). Nonalcoholic fatty liver disease promotes hepatocellular carcinoma through direct and indirect effects on hepatocytes. FEBS J..

[31] Huber Y., Labenz C., Michel M., Wörns M.-A., Galle P.R., Kostev K., Schattenberg J.M. (2020). Tumor Incidence in Patients with Non-Alcoholic Fatty Liver Disease. Dtsch. Ärzteblatt Int..

[32] Fukunaga S., Nakano D., Kawaguchi T., Eslam M., Ouchi A., Nagata T., Kuroki H., Kawata H., Abe H., Nouno R. (2021). Non-Obese MAFLD Is Associated with Colorectal Adenoma in Health Check Examinees: A Multicenter Retrospective Study. Int. J. Mol. Sci..

[33] Koskinas J., Gomatos I.P., Tiniakos D.G., Memos N., Boutsikou M., Garatzioti A., Archimandritis A., Betrosian A. (2008). Liver histology in ICU patients dying from sepsis: A clinico-pathological study. World J. Gastroenterol..

[34] Shaker M., Tabbaa A., Albeldawi M., Alkhouri N. (2014). Liver transplantation for nonalcoholic fatty liver disease: New challenges and new opportunities. World J. Gastroenterol..

[35] Wong R.J., Cheung R., Ahmed A. (2014). Nonalcoholic steatohepatitis is the most rapidly growing indication for liver transplantation in patients with hepatocellular carcinoma in the U.S. Hepatology.

[36] Wang X., Li J., Riaz D.R., Shi G., Liu C., Dai Y. (2014). Outcomes of liver transplantation for nonalcoholic steatohepatitis: A systematic review and meta-analysis. Clin. Gastroenterol. Hepatol..

[37] Hoppe S., von Loeffelholz C., Lock J.F., Doecke S., Sinn B.V., Rieger A., Malinowski M., Pfeiffer A.F.H., Neuhaus P., Stockmann M. (2015). Nonalcoholic steatohepatits and liver steatosis modify partial hepatectomy recovery. J. Investig. Surg..

[38] Stepanova M., Henry L., Garg R., Kalwaney S., Saab S., Younossi Z. (2015). Risk of de novo post-transplant type 2 diabetes in patients undergoing liver transplant for non-alcoholic steatohepatitis. BMC Gastroenterol..

[39] Golabi P., Otgonsuren M., de Avila L., Sayiner M., Rafiq N., Younossi Z.M. (2018). Components of metabolic syndrome increase the risk of mortality in nonalcoholic fatty liver disease (NAFLD). Medicine.

[40] Sommerfeld O., von Loeffelholz C., Diab M., Kiessling S., Doenst T., Bauer M., Sponholz C. (2020). Association between high dose catecholamine support and liver dysfunction following cardiac surgery. J. Card. Surg..

[41] Byrne C.D., Targher G. (2015). NAFLD: A multisystem disease. J. Hepatol..

[42] Paik J.M., Golabi P., Biswas R., Alqahtani S., Venkatesan C., Younossi Z.M. (2020). Nonalcoholic Fatty Liver Disease and Alcoholic Liver Disease are Major Drivers of Liver Mortality in the United States. Hepatol. Commun..

[43] Skyler J.S., Bakris G.L., Bonifacio E., Darsow T., Eckel R.H., Groop L., Groop P.-H., Handelsman Y., Insel R.A., Mathieu C. (2017). Differentiation of Diabetes by Pathophysiology, Natural History, and Prognosis. Diabetes.

[44] von Loeffelholz C., Coldewey S.M., Birkenfeld A.L. (2021). A Narrative Review on the Role of AMPK on De Novo Lipogenesis in Non-Alcoholic Fatty Liver Disease: Evidence from Human Studies. Cells.

[45] Lewis G.F., Carpentier A., Adeli K., Giacca A. (2002). Disordered fat storage and mobilization in the pathogenesis of insulin resistance and type 2 diabetes. Endocr. Rev..

[46] Bril F., Barb D., Portillo-Sanchez P., Biernacki D., Lomonaco R., Suman A., Weber M.H., Budd J.T., Lupi M.E., Cusi K. (2017). Metabolic and histological implications of intrahepatic triglyceride content in nonalcoholic fatty liver disease. Hepatology.

[47] Jonker J.T., de Laet C., Franco O.H., Peeters A., Mackenbach J., Nusselder W.J. (2006). Physical activity and life expectancy with and without diabetes: Life table analysis of the Framingham Heart Study. Diabetes Care.

[48] Sigal R.J., Kenny G.P., Wasserman D.H., Castaneda-Sceppa C., White R.D. (2006). Physical activity/exercise and type 2 diabetes: A consensus statement from the American Diabetes Association. Diabetes Care.

[49] Kirwan J.P., Sacks J., Nieuwoudt S. (2017). The essential role of exercise in the management of type 2 diabetes. Clevel. Clin. J. Med..

[50] Golabi P., Gerber L., Paik J.M., Deshpande R., de Avila L., Younossi Z.M. (2020). Contribution of sarcopenia and physical inactivity to mortality in people with non-alcoholic fatty liver disease. JHEP Rep..

[51] Tokushige K., Ikejima K., Ono M., Eguchi Y., Kamada Y., Itoh Y., Akuta N., Yoneda M., Iwasa M., Yoneda M. (2021). Evidence-based clinical practice guidelines for nonalcoholic fatty liver disease/nonalcoholic steatohepatitis 2020. J. Gastroenterol..

[52] Kelley D.E. (2005). Skeletal muscle fat oxidation: Timing and flexibility are everything. J. Clin. Investig..

[53] Corpeleijn E., Saris W.H.M., Blaak E.E. (2009). Metabolic flexibility in the development of insulin resistance and type 2 diabetes: Effects of lifestyle. Obes. Rev..

[54] Goodpaster B.H., Sparks L.M. (2017). Metabolic Flexibility in Health and Disease. Cell Metab..

[55] Smith R.L., Soeters M.R., Wüst R.C.I., Houtkooper R.H. (2018). Metabolic Flexibility as an Adaptation to Energy Resources and Requirements in Health and Disease. Endocr. Rev..

[56] Colberg S.R., Simoneau J.A., Thaete F.L., Kelley D.E. (1995). Skeletal muscle utilization of free fatty acids in women with visceral obesity. J. Clin. Investig..

[57] Samuel V.T., Shulman G.I. (2018). Nonalcoholic Fatty Liver Disease as a Nexus of Metabolic and Hepatic Diseases. Cell Metab..

[58] Petersen K.F., Shulman G.I. (2002). Pathogenesis of skeletal muscle insulin resistance in type 2 diabetes mellitus. Am. J. Cardiol..

[59] Ng J.M., Azuma K., Kelley C., Pencek R., Radikova Z., Laymon C., Price J., Goodpaster B.H., Kelley D.E. (2012). PET imaging reveals distinctive roles for different regional adipose tissue depots in systemic glucose metabolism in nonobese humans. Am. J. Physiol. Endocrinol. Metab..

[60] Haddad F., Adams G.R. (2002). Selected contribution: Acute cellular and molecular responses to resistance exercise. J. Appl. Physiol..

[61] Henriksen E.J. (2002). Invited review: Effects of acute exercise and exercise training on insulin resistance. J. Appl. Physiol..

[62] Zierath J.R. (2002). Invited review: Exercise training-induced changes in insulin signaling in skeletal muscle. J. Appl. Physiol..

[63] Sakamoto K., Goodyear L.J. (2002). Invited review: Intracellular signaling in contracting skeletal muscle. J. Appl. Physiol..

[64] Roden M. (2004). How free fatty acids inhibit glucose utilization in human skeletal muscle. News Physiol. Sci..

[65] Holloszy J.O. (2005). Exercise-induced increase in muscle insulin sensitivity. J. Appl. Physiol..

[66] Richter E.A., Hargreaves M. (2013). Exercise, GLUT4, and skeletal muscle glucose uptake. Physiol. Rev..

[67] Hegarty B.D., Turner N., Cooney G.J., Kraegen E.W. (2009). Insulin resistance and fuel homeostasis: The role of AMP-activated protein kinase. Acta Physiol..

[68] Petersen M.C., Shulman G.I. (2018). Mechanisms of Insulin Action and Insulin Resistance. Physiol. Rev..

[69] Herzig S., Shaw R.J. (2018). AMPK: Guardian of metabolism and mitochondrial homeostasis. Nat. Rev. Mol. Cell Biol..

[70] Nielsen S., Guo Z., Johnson C.M., Hensrud D.D., Jensen M.D. (2004). Splanchnic lipolysis in human obesity. J. Clin. Investig..

[71] Roden M. (2006). Mechanisms of Disease: Hepatic steatosis in type 2 diabetes—Pathogenesis and clinical relevance. Nat. Clin. Pract. Endocrinol. Metab..

[72] Roden M., Price T.B., Perseghin G., Petersen K.F., Rothman D.L., Cline G.W., Shulman G.I. (1996). Mechanism of free fatty acid-induced insulin resistance in humans. J. Clin. Investig..

[73] Abbasi F., McLaughlin T., Lamendola C., Reaven G.M. (2000). The relationship between glucose disposal in response to physiological hyperinsulinemia and basal glucose and free fatty acid concentrations in healthy volunteers. J. Clin. Endocrinol. Metab..

[74] Amati F., Dubé J.J., Alvarez-Carnero E., Edreira M.M., Chomentowski P., Coen P.M., Switzer G.E., Bickel P.E., Stefanovic-Racic M., Toledo F.G.S. (2011). Skeletal muscle triglycerides, diacylglycerols, and ceramides in insulin resistance: Another paradox in endurance-trained athletes?. Diabetes.

[75] Coen P.M., Menshikova E.V., Distefano G., Zheng D., Tanner C.J., Standley R.A., Helbling N.L., Dubis G.S., Ritov V.B., Xie H. (2015). Exercise and Weight Loss Improve Muscle Mitochondrial Respiration, Lipid Partitioning, and Insulin Sensitivity After Gastric Bypass Surgery. Diabetes.

[76] Chee C., Shannon C.E., Burns A., Selby A.L., Wilkinson D., Smith K., Greenhaff P.L., Stephens F.B. (2016). Relative Contribution of Intramyocellular Lipid to Whole-Body Fat Oxidation Is Reduced with Age but Subsarcolemmal Lipid Accumulation and Insulin Resistance Are Only Associated with Overweight Individuals. Diabetes.

[77] Lee H.-Y., Choi C.S., Birkenfeld A.L., Alves T.C., Jornayvaz F.R., Jurczak M.J., Zhang D., Woo D.K., Shadel G.S., Ladiges W. (2010). Targeted expression of catalase to mitochondria prevents age-associated reductions in mitochondrial function and insulin resistance. Cell Metab..

[78] Morino K., Petersen K.F., Dufour S., Befroy D., Frattini J., Shatzkes N., Neschen S., White M.F., Bilz S., Sono S. (2005). Reduced mitochondrial density and increased IRS-1 serine phosphorylation in muscle of insulin-resistant offspring of type 2 diabetic parents. J. Clin. Investig..

[79] Cleland P.J., Appleby G.J., Rattigan S., Clark M.G. (1989). Exercise-induced translocation of protein kinase C and production of diacylglycerol and phosphatidic acid in rat skeletal muscle in vivo. Relationship to changes in glucose transport. J. Biol. Chem..

[80] Ishizuka T., Cooper D.R., Hernandez H., Buckley D., Standaert M., Farese R.V. (1990). Effects of insulin on diacylglycerol-protein kinase C signaling in rat diaphragm and soleus muscles and relationship to glucose transport. Diabetes.

[81] You J.-S., Lincoln H.C., Kim C.-R., Frey J.W., Goodman C.A., Zhong X.-P., Hornberger T.A. (2014). The role of diacylglycerol kinase ζ and phosphatidic acid in the mechanical activation of mammalian target of rapamycin (mTOR) signaling and skeletal muscle hypertrophy. J. Biol. Chem..

[82] Koliaki C., Szendroedi J., Kaul K., Jelenik T., Nowotny P., Jankowiak F., Herder C., Carstensen M., Krausch M., Knoefel W.T. (2015). Adaptation of hepatic mitochondrial function in humans with non-alcoholic fatty liver is lost in steatohepatitis. Cell Metab..

[83] Szendroedi J., Roden M. (2008). Mitochondrial fitness and insulin sensitivity in humans. Diabetologia.

[84] Boushel R., Lundby C., Qvortrup K., Sahlin K. (2014). Mitochondrial plasticity with exercise training and extreme environments. Exerc. Sport Sci. Rev..

[85] Steenberg D.E., Jørgensen N.B., Birk J.B., Sjøberg K.A., Kiens B., Richter E.A., Wojtaszewski J.F.P. (2019). Exercise training reduces the insulin-sensitizing effect of a single bout of exercise in human skeletal muscle. J. Physiol..

[86] Lundby A.-K.M., Jacobs R.A., Gehrig S., de Leur J., Hauser M., Bonne T.C., Flück D., Dandanell S., Kirk N., Kaech A. (2018). Exercise training increases skeletal muscle mitochondrial volume density by enlargement of existing mitochondria and not de novo biogenesis. Acta Physiol..

[87] Essén-Gustavsson B., Tesch P.A. (1990). Glycogen and triglyceride utilization in relation to muscle metabolic characteristics in men performing heavy-resistance exercise. Eur. J. Appl. Physiol. Occup. Physiol..

[88] Koopman R., Manders R.J.F., Jonkers R.A.M., Hul G.B.J., Kuipers H., van Loon L.J.C. (2006). Intramyocellular lipid and glycogen content are reduced following resistance exercise in untrained healthy males. Eur. J. Appl. Physiol..

[89] van Loon L.J.C., Goodpaster B.H. (2006). Increased intramuscular lipid storage in the insulin-resistant and endurance-trained state. Pflug. Arch..

[90] Hoppeler H., Howald H., Conley K., Lindstedt S.L., Claassen H., Vock P., Weibel E.R. (1985). Endurance training in humans: Aerobic capacity and structure of skeletal muscle. J. Appl. Physiol..

[91] Dubé J.J., Amati F., Stefanovic-Racic M., Toledo F.G.S., Sauers S.E., Goodpaster B.H. (2008). Exercise-induced alterations in intramyocellular lipids and insulin resistance: The athlete’s paradox revisited. Am. J. Physiol. Endocrinol. Metab..

[92] Bogardus C., Lillioja S., Stone K., Mott D. (1984). Correlation between muscle glycogen synthase activity and in vivo insulin action in man. J. Clin. Investig..

[93] Ivy J.L. (1991). Muscle glycogen synthesis before and after exercise. Sports Med..

[94] Acheson K.J., Schutz Y., Bessard T., Anantharaman K., Flatt J.P., Jéquier E. (1988). Glycogen storage capacity and de novo lipogenesis during massive carbohydrate overfeeding in man. Am. J. Clin. Nutr..

[95] Ivy J.L. (2004). Regulation of muscle glycogen repletion, muscle protein synthesis and repair following exercise. J. Sports Sci. Med..

[96] Stephens F.B., Tsintzas K. (2018). Metabolic and molecular changes associated with the increased skeletal muscle insulin action 24–48 h after exercise in young and old humans. Biochem. Soc. Trans..

[97] de Bock K., Richter E.A., Russell A.P., Eijnde B.O., Derave W., Ramaekers M., Koninckx E., Léger B., Verhaeghe J., Hespel P. (2005). Exercise in the fasted state facilitates fibre type-specific intramyocellular lipid breakdown and stimulates glycogen resynthesis in humans. J. Physiol..

[98] Gaster M., Vach W., Beck-Nielsen H., Schrøder H.D. (2002). GLUT4 expression at the plasma membrane is related to fibre volume in human skeletal muscle fibres. APMIS.

[99] Kirwan J.P., Del Aguila L.F. (2003). Insulin signalling, exercise and cellular integrity. Biochem. Soc. Trans..

[100] King D.S., Feltmeyer T.L., Baldus P.J., Sharp R.L., Nespor J. (1993). Effects of eccentric exercise on insulin secretion and action in humans. J. Appl. Physiol..

[101] Flockhart M., Nilsson L.C., Tais S., Ekblom B., Apró W., Larsen F.J. (2021). Excessive exercise training causes mitochondrial functional impairment and decreases glucose tolerance in healthy volunteers. Cell Metab..

[102] Egan B., Zierath J.R. (2013). Exercise metabolism and the molecular regulation of skeletal muscle adaptation. Cell Metab..

[103] Hoffman N.J., Parker B.L., Chaudhuri R., Fisher-Wellman K.H., Kleinert M., Humphrey S.J., Yang P., Holliday M., Trefely S., Fazakerley D.J. (2015). Global Phosphoproteomic Analysis of Human Skeletal Muscle Reveals a Network of Exercise-Regulated Kinases and AMPK Substrates. Cell Metab..

[104] Larson-Meyer D.E., Newcomer B.R., Hunter G.R. (2002). Influence of endurance running and recovery diet on intramyocellular lipid content in women: A 1H NMR study. Am. J. Physiol. Endocrinol. Metab..

[105] Zderic T.W., Davidson C.J., Schenk S., Byerley L.O., Coyle E.F. (2004). High-fat diet elevates resting intramuscular triglyceride concentration and whole-body lipolysis during exercise. Am. J. Physiol. Endocrinol. Metab..

[106] Hu G., Eriksson J., Barengo N.C., Lakka T.A., Valle T.T., Nissinen A., Jousilahti P., Tuomilehto J. (2004). Occupational, commuting, and leisure-time physical activity in relation to total and cardiovascular mortality among Finnish subjects with type 2 diabetes. Circulation.

[107] Lindström J., Louheranta A., Mannelin M., Rastas M., Salminen V., Eriksson J., Uusitupa M., Tuomilehto J. (2003). The Finnish Diabetes Prevention Study (DPS): Lifestyle intervention and 3-year results on diet and physical activity. Diabetes Care.

[108] Knowler W.C., Barrett-Connor E., Fowler S.E., Hamman R.F., Lachin J.M., Walker E.A., Nathan D.M. (2002). Reduction in the incidence of type 2 diabetes with lifestyle intervention or metformin. N. Engl. J. Med..

[109] Roumen C., Corpeleijn E., Feskens E.J.M., Mensink M., Saris W.H.M., Blaak E.E. (2008). Impact of 3-year lifestyle intervention on postprandial glucose metabolism: The SLIM study. Diabet. Med..

[110] den Boer A.T., Herraets I.J.T., Stegen J., Roumen C., Corpeleijn E., Schaper N.C., Feskens E., Blaak E.E. (2013). Prevention of the metabolic syndrome in IGT subjects in a lifestyle intervention: Results from the SLIM study. Nutr. Metab. Cardiovasc. Dis..

[111] Wing R.R., Bolin P., Brancati F.L., Bray G.A., Clark J.M., Coday M., Crow R.S., Curtis J.M., Egan C.M., Espeland M.A. (2013). Cardiovascular effects of intensive lifestyle intervention in type 2 diabetes. N. Engl. J. Med..

[112] Gregg E., Jakicic J., Blackburn G., Bloomquist P., Bray G., Clark J., Coday M., Curtis J., Egan C., Evans M. (2016). Association of the magnitude of weight loss and changes in physical fitness with long-term cardiovascular disease outcomes in overweight or obese people with type 2 diabetes: A post-hoc analysis of the Look AHEAD randomised clinical trial. Lancet Diabetes Endocrinol..

[113] Sigal R.J., Kenny G.P., Wasserman D.H., Castaneda-Sceppa C. (2004). Physical activity/exercise and type 2 diabetes. Diabetes Care.

[114] Davies K.A.B., Sprung V.S., Norman J.A., Thompson A., Mitchell K.L., Halford J.C.G., Harrold J.A., Wilding J.P.H., Kemp G.J., Cuthbertson D.J. (2018). Short-term decreased physical activity with increased sedentary behaviour causes metabolic derangements and altered body composition: Effects in individuals with and without a first-degree relative with type 2 diabetes. Diabetologia.

[115] Romero-Gómez M., Zelber-Sagi S., Trenell M. (2017). Treatment of NAFLD with diet, physical activity and exercise. J. Hepatol..

[116] Sargeant J.A., Gray L.J., Bodicoat D.H., Willis S.A., Stensel D.J., Nimmo M.A., Aithal G.P., King J.A. (2018). The effect of exercise training on intrahepatic triglyceride and hepatic insulin sensitivity: A systematic review and meta-analysis. Obes. Rev..

[117] Ueno T., Sugawara H., Sujaku K., Hashimoto O., Tsuji R., Tamaki S., Torimura T., Inuzuka S., Sata M., Tanikawa K. (1997). Therapeutic effects of restricted diet and exercise in obese patients with fatty liver. J. Hepatol..

[118] Slentz C.A., Bateman L.A., Willis L.H., Shields A.T., Tanner C.J., Piner L.W., Hawk V.H., Muehlbauer M.J., Samsa G.P., Nelson R.C. (2011). Effects of aerobic vs. resistance training on visceral and liver fat stores, liver enzymes, and insulin resistance by HOMA in overweight adults from STRRIDE AT/RT. Am. J. Physiol. Endocrinol. Metab..

[119] Fealy C.E., Haus J.M., Solomon T.P.J., Pagadala M., Flask C.A., McCullough A.J., Kirwan J.P. (2012). Short-term exercise reduces markers of hepatocyte apoptosis in nonalcoholic fatty liver disease. J. Appl. Physiol..

[120] Bacchi E., Negri C., Targher G., Faccioli N., Lanza M., Zoppini G., Zanolin E., Schena F., Bonora E., Moghetti P. (2013). Both resistance training and aerobic training reduce hepatic fat content in type 2 diabetic subjects with nonalcoholic fatty liver disease (the RAED2 Randomized Trial). Hepatology.

[121] Haus J.M., Solomon T.P.J., Kelly K.R., Fealy C.E., Kullman E.L., Scelsi A.R., Lu L., Pagadala M.R., McCullough A.J., Flask C.A. (2013). Improved hepatic lipid composition following short-term exercise in nonalcoholic fatty liver disease. J. Clin. Endocrinol. Metab..

[122] Khaoshbaten M., Gholami N., Sokhtehzari S., Monazami A.H., Nejad M.R. (2013). The effect of an aerobic exercise on serum level of liver enzymes and liver echogenicity in patients with non alcoholic fatty liver disease. Gastroenterol. Hepatol. Bed Bench.

[123] Yoshimura E., Kumahara H., Tobina T., Matsuda T., Ayabe M., Kiyonaga A., Anzai K., Higaki Y., Tanaka H. (2014). Lifestyle intervention involving calorie restriction with or without aerobic exercise training improves liver fat in adults with visceral adiposity. J. Obes..

[124] Oh S., Shida T., Yamagishi K., Tanaka K., So R., Tsujimoto T., Shoda J. (2015). Moderate to vigorous physical activity volume is an important factor for managing nonalcoholic fatty liver disease: A retrospective study. Hepatology.

[125] Shamsoddini A., Sobhani V., Chehreh M.E.G., Alavian S.M., Zaree A. (2015). Effect of Aerobic and Resistance Exercise Training on Liver Enzymes and Hepatic Fat in Iranian Men with Nonalcoholic Fatty Liver Disease. Hepat. Mon..

[126] Takahashi A., Abe K., Usami K., Imaizumi H., Hayashi M., Okai K., Kanno Y., Tanji N., Watanabe H., Ohira H. (2015). Simple Resistance Exercise helps Patients with Non-alcoholic Fatty Liver Disease. Int. J. Sports Med..

[127] Charatcharoenwitthaya P., Kuljiratitikal K., Aksornchanya O., Chaiyasoot K., Bandidniyamanon W., Charatcharoenwitthaya N. (2021). Moderate-Intensity Aerobic vs. Resistance Exercise and Dietary Modification in Patients with Nonalcoholic Fatty Liver Disease: A Randomized Clinical Trial. Clin. Transl. Gastroenterol..

[128] Johnson N.A., Sachinwalla T., Walton D.W., Smith K., Armstrong A., Thompson M.W., George J. (2009). Aerobic exercise training reduces hepatic and visceral lipids in obese individuals without weight loss. Hepatology.

[129] Hallsworth K., Fattakhova G., Hollingsworth K.G., Thoma C., Moore S., Taylor R., Day C.P., Trenell M.I. (2011). Resistance exercise reduces liver fat and its mediators in non-alcoholic fatty liver disease independent of weight loss. Gut.

[130] Lee S., Bacha F., Hannon T., Kuk J.L., Boesch C., Arslanian S. (2012). Effects of aerobic versus resistance exercise without caloric restriction on abdominal fat, intrahepatic lipid, and insulin sensitivity in obese adolescent boys: A randomized, controlled trial. Diabetes.

[131] Sullivan S., Kirk E.P., Mittendorfer B., Patterson B.W., Klein S. (2012). Randomized trial of exercise effect on intrahepatic triglyceride content and lipid kinetics in nonalcoholic fatty liver disease. Hepatology.

[132] Zelber-Sagi S., Buch A., Yeshua H., Vaisman N., Webb M., Harari G., Kis O., Fliss-Isakov N., Izkhakov E., Halpern Z. (2014). Effect of resistance training on non-alcoholic fatty-liver disease a randomized-clinical trial. World J. Gastroenterol..

[133] Hallsworth K., Thoma C., Hollingsworth K.G., Cassidy S., Anstee Q.M., Day C.P., Trenell M.I. (2015). Modified high-intensity interval training reduces liver fat and improves cardiac function in non-alcoholic fatty liver disease: A randomized controlled trial. Clin. Sci..

[134] Keating S.E., Hackett D.A., Parker H.M., O’Connor H.T., Gerofi J.A., Sainsbury A., Baker M.K., Chuter V.H., Caterson I.D., George J. (2015). Effect of aerobic exercise training dose on liver fat and visceral adiposity. J. Hepatol..

[135] Cuthbertson D.J., Shojaee-Moradie F., Sprung V.S., Jones H., Pugh C.J.A., Richardson P., Kemp G.J., Barrett M., Jackson N.C., Thomas E.L. (2016). Dissociation between exercise-induced reduction in liver fat and changes in hepatic and peripheral glucose homoeostasis in obese patients with non-alcoholic fatty liver disease. Clin. Sci..

[136] Zhang H.-J., He J., Pan L.-L., Ma Z.-M., Han C.-K., Chen C.-S., Chen Z., Han H.-W., Chen S., Sun Q. (2016). Effects of Moderate and Vigorous Exercise on Nonalcoholic Fatty Liver Disease: A Randomized Clinical Trial. JAMA Intern. Med..

[137] Houghton D., Thoma C., Hallsworth K., Cassidy S., Hardy T., Burt A.D., Tiniakos D., Hollingsworth K.G., Taylor R., Day C.P. (2017). Exercise Reduces Liver Lipids and Visceral Adiposity in Patients with Nonalcoholic Steatohepatitis in a Randomized Controlled Trial. Clin. Gastroenterol. Hepatol..

[138] Keating S.E., Hackett D.A., George J., Johnson N.A. (2012). Exercise and non-alcoholic fatty liver disease: A systematic review and meta-analysis. J. Hepatol..

[139] von Loeffelholz C., Jahreis G. (2005). Einfluss von Widerstandstraining auf Parameter des Glukosestoffwechsels bei Gesunden, Typ-2-Diabetikern und Individuen mit Anzeichen einer Insulinresistenz. Aktuelle Ernährungsmedizin.

[140] Pugh C.J.A., Sprung V.S., Jones H., Richardson P., Shojaee-Moradie F., Umpleby A.M., Green D.J., Cable N.T., Trenell M.I., Kemp G.J. (2016). Exercise-induced improvements in liver fat and endothelial function are not sustained 12 months following cessation of exercise supervision in nonalcoholic fatty liver disease. Int. J. Obes..

[141] von Loeffelholz C., Birkenfeld A., Feingold K.R., Anawalt B., Boyce A., Chrousos G., de Herder W.W., Dhatariya K., Dungan K., Hershman J.M., Hofland J., Kalra S., Kaltsas G., Koch C., Kopp P., Korbonits M., Kovacs C.S., Kuohung W., Laferrère B., Levy M., McGee E.A., McLachlan R., Morley J.E., New M., Purnell J., Sahay R., Singer F., Sperling M.A., Stratakis C.A., Trence D.L., Wilson D.P. (2018). Endotext: The Role of Non-Exercise Activity Thermogenesis in Human Obesity.

[142] Dasgupta K., Chan C., Da Costa D., Pilote L., de Civita M., Ross N., Strachan I., Sigal R., Joseph L. (2007). Walking behaviour and glycemic control in type 2 diabetes: Seasonal and gender differences—Study design and methods. Cardiovasc. Diabetol..

[143] Hawley J.A., Gibala M.J. (2009). Exercise intensity and insulin sensitivity: How low can you go?. Diabetologia.

[144] Chung N., Park M.-Y., Kim J., Park H.-Y., Hwang H., Lee C.-H., Han J.-S., So J., Park J., Lim K. (2018). Non-exercise activity thermogenesis (NEAT): A component of total daily energy expenditure. J. Exerc. Nutr. Biochem..

[145] Silva A.M., Júdice P.B., Carraça E.V., King N., Teixeira P.J., Sardinha L.B. (2018). What is the effect of diet and/or exercise interventions on behavioural compensation in non-exercise physical activity and related energy expenditure of free-living adults? A systematic review. Br. J. Nutr..

[146] Alahmadi M.A., Hills A.P., King N.A., Byrne N.M. (2011). Exercise intensity influences nonexercise activity thermogenesis in overweight and obese adults. Med. Sci. Sports Exerc..

[147] Chiu C.-H., Chen C.-H., Wu M.-H., Ding Y.-F. (2020). Nonexercise Activity Thermogenesis-Induced Energy Shortage Improves Postprandial Lipemia and Fat Oxidation. Life.

